# Association Between High-Sensitivity C-Reactive Protein (hs-CRP) Levels With Lipids and Micronutrients

**DOI:** 10.7759/cureus.67268

**Published:** 2024-08-20

**Authors:** Hari K Krishnamurthy, Swarnkumar Reddy, Vasanth Jayaraman, Karthik Krishna, Qi Song, Tianhao Wang, Kang Bei, John J Rajasekaran

**Affiliations:** 1 Biomedical Engineering, Vibrant America LLC, San Carlos, USA

**Keywords:** systemic inflammation, vitamins, cholesterol, lipids, micronutrients, hs-crp

## Abstract

Background and aims: Elevated high-sensitivity C-reactive protein (hs-CRP) levels are associated with an increased risk of cardiovascular disease, indicating systemic inflammation. Abnormal lipid levels and deficiencies in certain vitamins and minerals could also contribute to elevated hs-CRP levels. By broadly looking at the cross-correlations between inflammatory, lipid, and micronutrient markers, we aim to highlight the key associations at the serological levels.

Methods: A retrospective analysis was conducted on 1,014 free-living individuals who tested for cardiovascular and micronutrient panels along with hs-CRP at Vibrant America Clinical Laboratory.

Results and conclusion: Based on parametric t-tests, significant variations between the sexes (Ma1) were observed for cholesterol, high-density lipoprotein (HDL), triglycerides, vitamin A, vitamin D3, serum copper, and valine. Pearson’s correlation showed a high-significant positive correlation between hs-CRP and triglycerides, folate, serum copper, and manganese.

## Introduction

Systemic inflammation refers to a complex physiological response to harmful stimuli such as infection, trauma, surgery, or malignancy, involving a cascade of inflammatory mediators and immune cell activation [1]. This inflammation is critical in the resolution of these conditions. However, chronic inflammation, characterized by persistent immune activation, can lead to tissue damage and contribute to the pathogenesis of various diseases, including cardiovascular disease (CVD), diabetes, and cancer [2]. Inflammation is essential for healing in the short term but can be a factor for disease progression when it is chronic. The treatment and management options available for chronic inflammation depend on the underlying cause and may involve medication and lifestyle changes for improving care coordination and communication to advance the control of chronic inflammation and improve outcomes [3].

High-sensitivity C-reactive protein (hs-CRP) is an established marker of systemic inflammation and a predictor of future cardiac events. Elevated hs-CRP levels are associated with increased cardiovascular risk in both the general population and individuals with chronic kidney disease. Prospective studies have demonstrated that hs-CRP can independently predict the risk of coronary heart disease, with a two- to fourfold increased risk observed in patients with type 2 diabetes. The early detection of elevated levels of hs-CRP and identification of factors associated with increasing hs-CRP play a crucial role in preventing severe CVD and other related vascular disorders [4,5].

Micronutrients, including vitamins and minerals, are important in maintaining optimal health and preventing chronic diseases. In recent years, research has focused on understanding the effects of micronutrients on serum hs-CRP, an important biomarker of inflammation that is associated with increased risk of chronic diseases such as CVD, diabetes, and cancer [6]. Several reports have shown that certain micronutrients have anti-inflammatory effects and may help reduce hs-CRP levels. For example, folate and vitamin B12 have been found to have inverse associations with hs-CRP levels in several studies [7]. These micronutrients are involved in the metabolism of homocysteine. Vitamin D is another key micronutrient that has been found to have anti-inflammatory effects, and studies have shown that low levels of vitamin D are associated with higher hs-CRP levels [8]. Other micronutrients such as magnesium, zinc, and selenium have also been found to have potential anti-inflammatory effects and may help reduce hs-CRP levels. However, the evidence for these micronutrients is less consistent, and intense research is needed to understand their effects on hs-CRP fully [9].

Given the associations of hs-CRP with cardiac events and its association with micronutrients, we investigated the cross-correlation between these markers. Studying the association of serum hs-CRP with lipid markers and micronutrients can provide valuable insights into the relationship between inflammation nutrient status and CVD risk.

## Materials and methods

Study population

The study population was selected from the subjects who have been addressed to the Vibrant America Clinical Laboratory for testing serum levels of hs-CRP along with serum levels of vital lipids and micronutrients. The retrospective analysis was completed using the deidentified clinical data and test results from a total of 1,014 subjects, and hence the study was exempted from the formal ethical review by Western IRB (Washington, USA IRB no - #1-1098539-1).

hs-CRP

Serum hs-CRP levels were measured using a particle-enhanced immunoturbidimetric method, which measures the agglutinates of hs-CRP with latex particles coated with anti-CRP monoclonal antibodies. The concentration of hs-CRP is measured turbidimetrically on Cobas c 311 (Roche Diagnostics, Indianapolis, IN) analyzers. The functional sensitivity is the lowest hs-CRP concentration that can be reproducibly measured with an interassay coefficient of variation of < 10%.

Cardiovascular markers

Blood samples were processed for the separation of serum and analyzed for serum levels of total cholesterol, low-density lipoprotein (LDL), high-density lipoprotein (HDL), and triglycerides. Total cholesterol was measured by the cholesterol dehydrogenase method via the AU680 analyzer (Beckman Coulter Diagnostics, Brea, CA). Serum levels of LDL, HDL, and triglycerides were measured by an enzymatic colorimetric method using the Beckman Coulter AU680 analyzer.

Micronutrient panel

Serum levels of micronutrients were determined using a TQ-XS Tandem mass spectrometer (Waters Corporation, Milford, MA) coupled with LCMS, Waters GC-MS, and Perkin Elmer NexION ICP-MS using standard laboratory protocols.

Statistical analysis

The processing of clinical data from deidentified subjects was performed via Java for Windows version 1.8.161, and statistical analysis was performed using GraphPad Prism version 7.00 (Windows; GraphPad Software, San Diego, CA). The cross-sectional study aimed to examine the possible association between hs-CRP with lipids and micronutrients. Serum hs-CRP was used as a categorical variable and was divided into categories: < 0.5, 0.51-3.0, 3.01-10, 10.1-20, and > 20.00 mg/L. The lipids and micronutrients were used as continuous variables. Pearson’s correlation analysis was done to find a probable correlation between variables with significance set at p < 0.005.

## Results

The study comprised 1,014 individuals (447 males and 567 females), with a mean age of 51.2 ± 15.4 and 50.2 ± 14.3, respectively. The association of serum hs-CRP with lipid and micronutrient variables for males and females is listed in Table 1. A significant difference was observed between genders in serum hs-CRP. Cholesterol, HDL, and triglycerides exhibited a significant difference between males and females. Females were observed to have higher levels of serum cholesterol, LDL, and HDL. Among micronutrient variables, vitamin A, vitamin B6, vitamin C, vitamin D3, vitamin E, calcium, and valine had significant differences between males and females.

**Table 1:** Status of high-sensitivity C-reactive protein and nutritional variables in the study subjects

	Mean (95%)			P-value
	Male (n=447)	Female (n=567)	All (n=1014)	
hs-CRP (mg/L)	2.138 (0.15-66.4)	2.77 (0.15-46.2)	2.494 (0.15-66.4)	0.0286
Serum lipids				
Cholesterol (mg/dL)	188.2 (92-314)	200.80 (101-423)	195.2 (92-423)	<0.0001
Low-Density Lipoprotein (mg/dL)	127.8 (37-266)	128.90 (32-354)	128.4 (32-354)	0.6722
High-Density Lipoprotein (mg/dL)	51.17 (23-104)	62.77 (27-151)	57.66 (23-151)	<0.0001
Triglycerides (mg/dL)	107.9 (29-382)	97.64 (25-398)	102.2 (25-398)	0.0034
Serum micronutrients				
Vitamin A (mcg/dL)	75.11 (17.3-148.5)	68.78 (19.8-151.5)	71.57 (17.3-151.5)	<0.0001
Vitamin B1 (nmol/L)	18.92 (0.39-136.8)	19.25 (0.85-94.5)	19.1 (0.395-136.8)	0.7273
Vitamin B12 (pg/mL)	715.1 (196-1974)	725.40 (205.1-1980)	720.9 (196-1980)	0.6321
Vitamin B2 (mcg/L)	24.18 (2.4-227.2)	24.27 (2.3-222.5)	24.23 (2.3-227.2)	0.9515
Vitamin B3 (ng/mL)	18.53 (0.9-66.3)	18.19 (1.5-110.2)	18.34 (0.99-110.2)	0.5089
Vitamin B5 (mcg/L)	120.5 (4.9-482.2)	110.30 (13.5-448.7)	114.8 (4.9-482.2)	0.0674
Vitamin B6 (ng/mL)	16.52 (1.4-236.8)	14.12 (1.1-165.8)	15.18 (1.1-236.8)	0.0222
Vitamin C (mg/dL)	0.4211 (0.06-2.6)	0.45 (0.05-3.7)	0.43 (0.05-3.7)	0.0351
Vitamin D, 25-OH (ng/mL)	47.31 (9.8-145.3)	47.82 (8.8-136)	47.59 (8.8-145.3)	0.6964
Vitamin D3 (ng/mL)	0.8598 (0.28-1.8)	0.93 (0.27-2.0)	0.9 (0.27-2.0)	<0.0001
Vitamin E (mg/L)	13.82 (4.6-35.4)	14.43 (4.1-37.3)	14.16 (4.1-37.3)	0.0389
Vitamin K1 (ng/mL)	1.103 (0.01-7.3)	1.13 (2.6-20)	1.12 (0.01-10.4)	0.6451
Vitamin K2 (ng/mL)	0.6361 (0.016-7.9)	0.74 (0.02-11.2)	0.694 (0.01-11.2)	0.0959
Folate (ng/mL)	13.3 (2.8-19.9)	13.33 (2.6-20)	13.32 (2.6-20)	0.8993
Calcium (mg/dL)	9.736 (8.7-11.6)	9.65 (7.9-11.4)	9.68 (7.9-11.6)	0.0012
Copper Serum (mcg/mL)	5.704 (0.62-14.8)	5.79 (0.6-14.9)	1.03 (0.22-7.0)	<0.0001
Iron (ug/dL)	107.8 (18.7-254)	104.10 (25.3-367.2)	105.8 (18.7-367.2)	0.1319
Magnesium (mg/dL)	2.187 (1.6-2.7)	2.17 (1.0-2.9)	2.17 (1.0-2.9)	0.1077
Manganese (ng/mL)	0.787 (0.24-9.0)	0.78 (0.24-6.8)	0.781 (0.2-9.0)	0.7473
Potassium (mmol/L)	4.529 (3.3-6.2)	4.53 (3.5-6)	4.52 (3.3-6.2)	0.9420
Selenium (ng/mL)	141.5 (43.0-357.1)	143.20 (71.5-360.5)	142.4 (43.0-360.5)	0.3521
Serine (nmol/mL)	147.6 (33.8-277.5)	149 (59.7-293)	148.4 (33.8-293)	0.5248
Sodium (mmol/L)	140.1 (129-159)	141.30 (133-154)	140.8 (97-159)	0.2725
Zinc (mcg/mL)	0.720 (0.18-2.0)	0.70 (0.34-2.7)	0.71 (0.18-2.7)	0.1568
Arginine (nmol/mL)	125.4 (43.0-299.2)	125.70 (42.2-280.2)	125.5 (42.2-299.2)	0.8938
Asparagine (nmol/mL)	53.5 (18.0-113.5)	53.16 (21.5-113.1)	53.31 (18.0-113.5)	0.6486
Carnitine (nmol/mL)	27.15 (9.9-50.5)	27.03 (7.9-99.1)	27.08 (7.9-99.1)	0.7945
Choline (nmol/mL)	15.43 (2.4-39.6)	14.67 (1.0-36.6)	15 (1.0-39.6)	0.0275
Chromium (ng/mL)	0.271 (0.013-2.96)	0.28 (0.011-2.81)	0.276 (0.01-2.9)	0.5254
Citrulline (nmol/mL)	1.714 (0.009-4.85)	1.75 (0.34-4.9)	30.11 (10.1-74.9)	0.7824
Cysteine (nmol/mL)	18.97 (1.31-54.0)	19.76 (1.4-49.1)	19.41 (1.3-54.0)	0.1587
Isoleucine (nmol/mL)	54.96 (21.8-152.3)	56.42 (16.5-148.7)	55.78 (16.5-152.3)	0.2788
Leucine (nmol/mL)	161.5 (44.5-249.7)	159.80 (44.3-249.8)	160.6 (44.3-249.8)	0.4766
Valine (nmol/mL)	263.2 (116.7-442.7)	233.80 (62.0-475.6)	246.8 (62-475.6)	<0.0001

Lipid variability with hs-CRP

**Figure 1:**
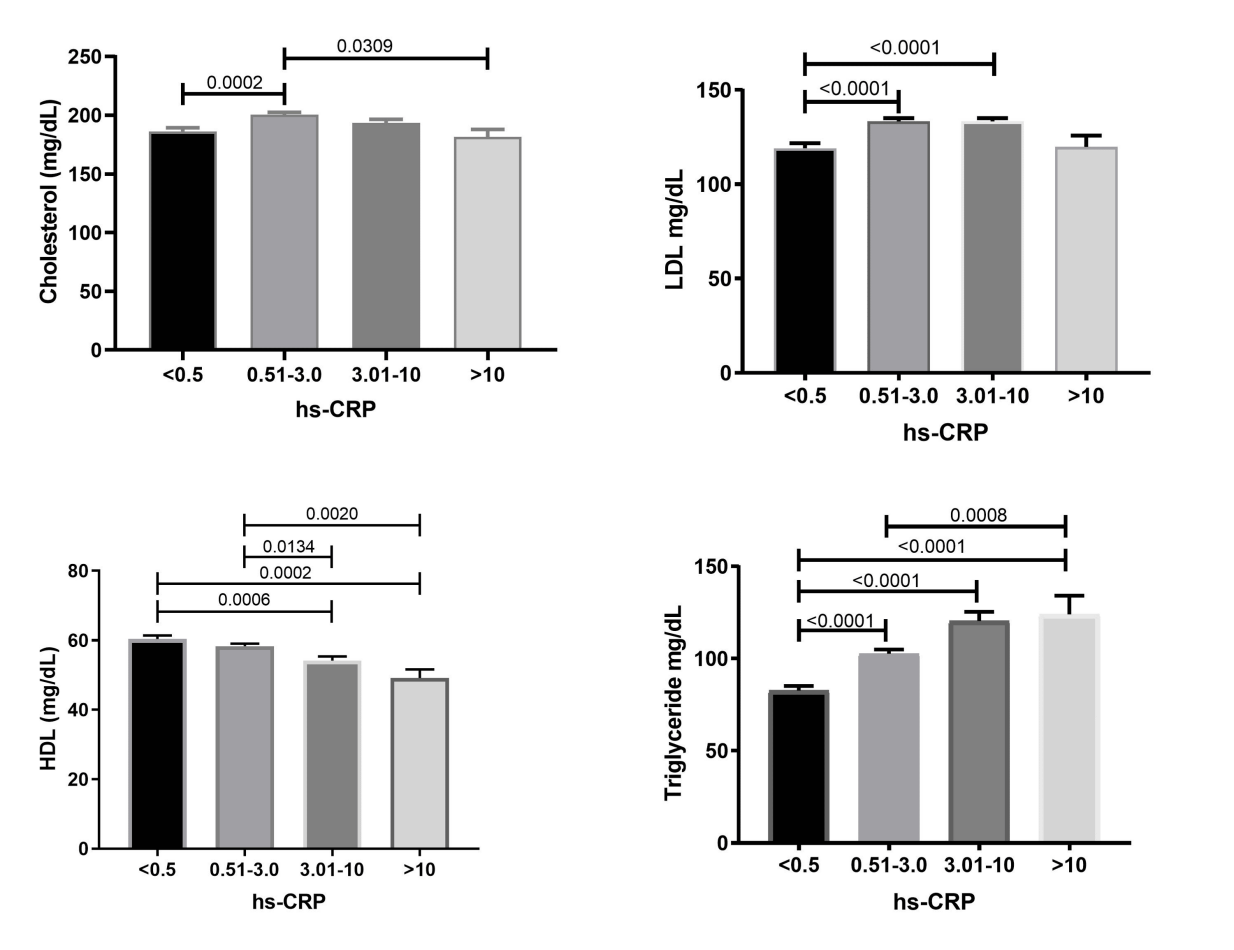
Mean (95% CI) of serum lipids in ordered hs-CRP categories in the total population

The significance between varying quartiles of hs-CRP and lipid markers was analyzed through one-way ANOVA. The serum levels of cholesterol were significantly associated with increasing quartiles of hs-CRP, and the mean cholesterol value was found to increase with mild elevation in serum hs-CRP (0.5-3.0 mg/dL). Meanwhile, further elevation (3.01 - > 10 mg/dL) in serum hs-CRP increased mean cholesterol. A significant interaction was observed between mean cholesterol and serum hs-CRP quartiles (p < 0.0001). Serum levels of LDL were significantly associated with hs-CRP quartiles (p < 0.0001), and a pairwise evaluation exhibited statistically significant variation at hs-CRP concentrations between 0.5 and 10 mg/dL (Figure 1). Serum levels of HDL were significantly associated with increasing quartiles of hs-CRP, and a significant decline in mean HDL was observed with an increase in hs-CRP quartiles. Triglycerides were significantly associated with changes in serum hs-CRP quartiles. An increase in the mean concentration of triglycerides increased serum concentrations of hs-CRP quartiles.

Micronutrient variability with hs-CRP

**Figure 2:**
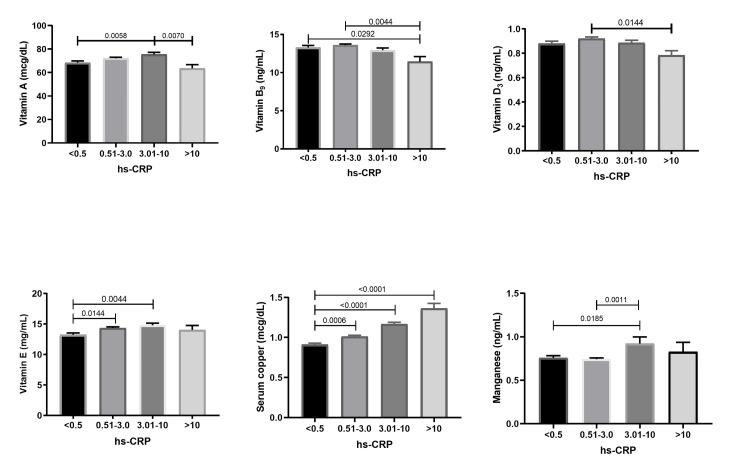
Mean (95% CI) of serum micronutrients in ordered hs-CRP categories in the total population

Levels of vitamin A were significantly associated with increasing concentrations of hs-CRP. The mean vitamin A was found to increase with an increase in hs-CRP to 10 mg/dL. Further increase in hs-CRP concentration resulted in a decrease in serum vitamin A. Serum folate was significantly associated with hs-CRP (p < 0.0038), and a significant decline in folate was observed with an increase in serum hs-CRP. The mean vitamin D3 concentrations were found to increase with the increase in a small amount of serum hs-CRP (0.51-3.0 mg/dL). Further, a constant decline in vitamin D3 was observed with an increase in hs-CRP beyond 3.1 mg/dL. Serum vitamin E was significantly associated with serum hs-CRP (p < 0.0043), and a significant increase in mean vitamin E concentration was observed with a small increase in hs-CRP (Figure 2).

A strong significant association was observed between hs-CRP and serum copper (p < 0.0001); with a constant increase in serum copper, an increase in serum hs-CRP was observed. In contrast, the overall tendency for manganese detected in serum was not clear. However, a significant association between serum hs-CRP and manganese. 

Pearson’s correlation analysis was conducted to assess the strength and direction of these relationships. The correlation analysis between serum hs-CRP and various lipid markers and micronutrients revealed several significant associations. Among, the lipid markers, cholesterol exhibited a weak negative correlation with hs-CRP (r = -0.08195, p = 0.009), indicating that higher cholesterol levels are associated with lower levels of hs-CRP. Similarly, LDL showed a weak negative correlation (r = -0.06203, p = 0.0483), suggesting that higher LDL levels are associated with lower hs-CRP levels. In contrast, HDL demonstrated a moderate negative correlation (r = -0.1386, p < 0.0001), indicating that higher HDL levels are associated with lower hs-CRP levels. Triglycerides exhibited a moderate positive correlation with hs-CRP (r = 0.1174, p = 0.0002), suggesting that higher triglyceride levels are associated with higher hs-CRP levels.

Among the micronutrients, folate showed a significant negative correlation with hs-CRP (r = -0.1061, p = 0.0007), indicating that higher folate levels are associated with lower hs-CRP levels. Copper demonstrated a strong positive correlation with hs-CRP (r = 0.2213, p < 0.0001), suggesting that higher copper levels are associated with higher hs-CRP levels. Vitamin C also showed a significant negative correlation with hs-CRP (r = -0.06283, p = 0.0455), indicating that higher vitamin C levels are associated with lower hs-CRP levels. Most of the other micronutrients did not show significant correlations with hs-CRP. The results of the correlation analysis are presented in Table 2, highlighting significant associations between hs-CRP and specific lipid markers and micronutrients.

**Table 2:** Pearson correlation coefficients between hs-CRP levels and various lipid markers and micronutrients **** p < 0.0001 (extremely significant), *** p < 0.001 (highly significant), ** p < 0.01 (very significant), * p < 0.05 (significant), ns not significant (p ≥ 0.05)

	r	P (<0.05)	P value summary
Lipid markers			
Cholesterol (mg/dL)	-0.08195	0.009	**
LDL (mg/dL)	-0.06203	0.0483	*
HDL (mg/dL)	-0.1386	<0.0001	****
Triglycerides (mg/dL)	0.1174	0.0002	***
Micronutrients			
Vitamin A (mcg/dL)	-0.03214	0.3066	ns
Vitamin B1 (nmol/dL)	-0.01818	0.5631	ns
Vitamin B12 (pg/mL)	-0.02504	0.4257	ns
Vitamin B2 (mcg/L)	0.01512	0.6306	ns
Vitamin B3 (ng/mL)	-0.00734	0.8153	ns
Vitamin B3 (pg/MM WBC)	-0.07888	0.012	*
Vitamin B5 (mcg/L)	-0.0249	0.4284	ns
Vitamin B6 (ng/mL)	-0.05283	0.0927	ns
Vitamin C (mg/dL)	-0.06283	0.0455	*
Vitamin DOH_25_(ng/mL)	-0.03471	0.2694	ns
Vitamin D3 (ng/mL)	-0.06762	0.0313	*
Vitamin D3 (pg/MM WBC)	-0.01889	0.5479	ns
Vitamin E (mg/L)	0.01568	0.618	ns
Vitamin K1 (ng/mL)	-0.03919	0.2125	ns
Vitamin K2 (ng/mL)	-0.01309	0.6773	ns
Folate (ng/mL)	-0.1061	0.0007	***
Calcium (mg/dL)	-0.05996	0.0563	ns
Copper (mcg/dL)	0.2213	<0.0001	****
Copper (ng/MM WBC)	-0.04229	0.1784	ns
Iron (µg/dL)	-0.03664	0.2438	ns
Magnesium (mg/dL)	-0.03148	0.3166	ns
Manganese (ng/mL)	0.08294	0.0082	**
Potassium (mmol/L)	-0.01186	0.7059	ns
Selenium (ng/mL)	-0.01135	0.7181	ns
Serine (ng/MM WBC)	0.03804	0.2262	ns
Sodium (mmol/L)	0.01024	0.7448	ns
Zinc (mcg/mL)	0.007811	0.8038	ns
Arginine (nmol/mL)	-0.05229	0.0961	ns
Asparagine (nmol/mL)	-0.00776	0.805	ns
Carnitine (nmol/mL)	-0.02122	0.4997	ns
Choline (nmol/mL)	-0.00596	0.8497	ns
Chromium (ng/mL)	-0.01797	0.5676	ns
Citrulline (nmol/mL)	-0.05244	0.0951	ns
Coenzyme Q10 (µg/mL)	-0.02466	0.4328	ns
Cysteine (nmol/mL)	-0.00239	0.9395	ns
Isoleucine (nmol/mL)	0.002503	0.9366	ns
Leuciune (nmol/mL)	0.0305	0.3319	ns
Valine (nmol/mL)	-0.02218	0.4805	ns

## Discussion

The present study details the association of various vital micronutrients - vitamins, minerals, and amino acids - along with lipid panel and serum levels of hs-CRP. The large sample size adds strength to our study, which is adequate to detect the possible associations between serum hs-CRP with lipids and micronutrients. Numerous reports have stated the importance of evaluating the micronutrient levels in patients with chronic disorders including prothrombotic, atherosclerotic, and inflammatory components [10]. The assessment of new markers such as micronutrients has been explored in the present study in addition to conventional lipid markers.

hs-CRP is an inflammatory marker that has been associated with several risk factors for cardiovascular disease, including dyslipidemia and micronutrient deficiencies. Dyslipidemia refers to abnormal lipid levels in the blood, including high levels of LDL cholesterol and HDL cholesterol. Elevated hs-CRP levels are associated with dyslipidemia, particularly high LDL cholesterol levels [11].

Micronutrient deficiencies, particularly of vitamins and minerals such as vitamin D, vitamin E, and selenium, have also been linked to elevated hs-CRP levels. Some studies suggest that these micronutrients may have anti-inflammatory effects and that deficiencies may contribute to chronic low-grade inflammation [12].

The study showed a significant association between cholesterol and increasing quartile levels of hs-CRP, where a rising trend was observed with an increase in hs-CRP from 0.5 to 3.0 mg/dL. LDL also exhibited a similar association with all quartile levels of hs-CRP. An increase in mean LDL has been observed with an increase in hs-CRP above 0.5 mg/dL, whereas a significant decrease in mean LDL was seen with an increase in hs-CRP above 10 mg/dL. The current result is in line with a report by Whelton et al. [13], where they reported serum cholesterol as a predictive marker of CHD when hs-CRP levels were < 2 mg/dL. They also reported hs-CRP as a weak or nonsignificant risk factor when elevated beyond 2 mg/dL. While LDL is a well-established risk factor for CVD, recent studies have shown that the size and count of LDL particles may be more important than LDL cholesterol concentration in atherogenesis. Inflammation plays a key role in the pathophysiology of atherosclerosis, and hs-CRP has been found to increase the expression of adhesion molecules and enhance LDL uptake by macrophages, contributing to the atherosclerotic process in vessels. Triglycerides exhibited a significant positive trend with increasing quartiles of serum hs-CRP. With these results, it can be hypothesized that a considerable increase in cholesterol and triglycerides from their normal limits potentiates inflammatory responses evidenced by the increase in hs-CRP levels. These inflammatory responses are induced by the infiltration of triglycerides-rich lipoproteins (TRL), VLDL, and chylomicron on the arterial wall sub-endothelial space [14]. The association between hs-CRP and TRL metabolism has been studied using stable isotope methodology. The study found that hs-CRP production rate was the main determinant of plasma hs-CRP levels, and there was a significant association between hs-CRP fractional catabolic rate (FCR) and TRL apoB-100 FCR, as well as between CRP pool size (PS) and TRL apoB-48 FCR, indicating linkage between hs-CRP and TRL metabolism.

The present study exhibited a strong negative correlation between serum hs-CRP and HDL and showed a significant negative trend with increasing quartile levels of hs-CRP [15]. Numerous prospective studies have reported a consistent inverse association between HDL and various systemic inflammatory markers. The reverse cholesterol transportation pathway is a well-known key factor of HDL metabolism, apart from this HDL is also involved in various other cellular functions including intercellular communication, anti-apoptotic, anti-inflammatory, anti-oxidative, and pro-vasodilatory activities. A study by Nunes et al. [16] on the association between treadmill exercise response and serum hs-CRP stated a similar inverse association between HDL and hs-CRP. They reported that a decrease in 1 mg/dL of HDL was expected to increase serum hs-CRP by 14.6%.

hs-CRP is a marker of inflammation that has been shown to affect endothelial function by decreasing endothelial nitric oxide synthase (eNOS) activity in endothelial cells and inhibiting endothelium-dependent nitric oxide (NO)-mediated vasodilation in vitro [17,18]. Elevated CRP levels have also been associated with hyperactivity of the endothelin-1 (ET-1) system, which is linked with cardiovascular disease development and progression. CRP has been shown to stimulate growth differentiation factor 15 (GDF15) expression in endothelial cells through p53, which can lead to endothelial dysfunction and atherosclerosis [19].

Furthermore, studies have implicated adverse effects of CRP on endothelial cell health and function, including decreased endothelial fibrinolytic capacity and endothelium-dependent vasodilation, which can contribute to cardiovascular disease development. Several studies have reported a significant association between hs-CRP and serum vitamin A (retinol), which is transported by a retinol-binding protein and assists in preventing systemic inflammation. Tuuminen et al. [6] represented vitamin A as a sensitive marker for inflammation and mild elevation in CRP may decrease about 40% of serum vitamin A with no further effect as CRP level increases beyond 15 mg/dL. This report agrees with our current results as the mean vitamin A concentration tends to decrease with an increase in hs-CRP > 10 mg/dL. A considerable number of reports have detailed about supplementation of B vitamin complexes for reducing serum CRP [20]. A recent report in 2021 by Zargarzadeh et al. [21] in their work on the impact of folic acid supplementation on CRP demonstrated a better reduction in CRP concentration after folic acid (Vit B9) treatment. Homocysteine decline through folic acid supplementation acts as an antioxidant by NADPH oxidase regulation or by reducing oxidative stress (pathologic oxidative stress) which enhances the expression of inflammatory markers [21]. An inverse trend was observed between vitamin B9 with an increase in hs-CRP and a strong negative correlation between serum vitamin B9 and hs-CRP, indicating that the decrease in the serum folate induces oxidative stress and increases the serum hs-CRP [22].

Osteoporosis is a prevalent proxy for supplementation of vitamin D, immune cells such as dendritic cells and macrophages were known to produce the active form of vitamin D by expressing 1-a-hydroxylase which readily converts 25-hydroxyvitamin D to active 1.25-dihydroxyvitamin D [23]. The active form of vitamin D is shown to inhibit the production of inflammatory markers (IFN- γ, IL5, and IL2) and inhibits the synthesis of CRP by inhibiting the IL6. Several observational studies have shown the inverse association between vitamin D3 and hs-CRP. The present study exhibited a non-significant negative correlation between hs-CRP and vitamin D3, but the mean vitamin D3 concentrations were found to exhibit a significant decreasing trend with an increase in hs-CRP above 0.5 mg/dL. From these results, it can be deduced that a decrease in the dietary intake of vitamin D3 increases serum hs-CRP levels [24,25].

Vitamin E is one of the most common antioxidative supplements (Vit E, Vit C, carotenoids, Se, and Zn). Vitamin E triggers a direct anti-inflammatory effect by down-regulating pro-inflammatory IL-1β mediated by inhibition of 5-lipoxygenase, an enzyme that regulates inflammatory prostaglandin synthesis. The anti-inflammatory effect of vitamin E can also be brought by inhibiting the synthesis of proinflammatory transcription factor (NF-kB) or by reducing lipopolysaccharide-stimulated proinflammatory cytokine TNF-α [26]. The inflammation is promoted by both NF-kB-mediated activation and also by proinflammatory cytokines, so the anti-inflammatory effect of vitamin E acts via both direct anti-inflammatory and also in oxidative pathways. The formation of oxidized vitamin E has a pro-oxidative effect when supplemented alone, so it is usually combined with the hydrophilic antioxidant vitamin C [27]. Considerable studies have reported a positive association between vitamins and higher CRP levels. The present study also observed a non-significant positive correlation between hs-CRP and vitamin E. However the mean vitamin E concentrations were found to have a significant association with all quartiles of hs-CRP. A negligible increase in the mean vitamin E concentration was observed in the first quartile, and this supports the results of Schwab et al., who observed a positive association between vitamin E and CRP. The decrease in the mean vitamin E at higher hs-CRP concentrations might result from the pro-oxidative properties of vitamin E. The subjects were found to have a significant negative correlation with vitamin C, and this also accounts for the pro-oxidative effect of vitamin E.

Copper is a vital micronutrient and an acute phase reactant during inflammation. The serum copper is detected as a measure of copper bound to caeruloplasmin (Cp). Cp is an acute phase reactant and has the potential to inhibit oxidative reactants peroxide and superoxide. Several reports have demonstrated a significant positive association between CRP and copper levels in the blood [28,29]. Elevated levels of Cp can contribute to various inflammatory reactions and have pro-oxidative effects such as lipid peroxidation [29,30]. The results from the present study are also in line with previous observations as the serum copper was found to have a strong positive correlation with serum hs-CRP. Additionally, the mean serum copper concentrations were found to have a strong significance and a positive trend toward the increasing quartile of serum hs-CRP. The cardioprotective nature of Cp in protecting myocardial tissue against the effect of free radicals was proven. However, the elevated level of Cp in association with inflammatory markers was used for the determination of the future risk of cardiovascular events. Devanarayanan et al. [31] reported that the positive correlation between CRP and serum copper may be associated with the pathogenesis of schizophrenia, including the risk of cardiovascular disease. They also stated that prolonged inflammation results in disturbing cerebral blood flow and damages the micro-vascular system resulting in psychopathology [32].

All lipid markers exhibited significant variation with increasing hs-CRP levels, whereas among micronutrients vitamin A, vitamin D3, vitamin E, vitamin B3, serum copper, and manganese exhibited considerable significance with increasing hs-CRP levels. Pearson’s correlations suggest a strong negative correlation with HDL and a strong positive correlation with triglycerides. Folate exhibited a strong negative correlation, whereas serum copper and manganese exhibited a strong positive correlation with serum hs-CRP. This could be indicative of underlying inflammatory processes or nutritional imbalances. Micronutrients could thus be an important factor in modulating lipid markers and inflammation.

While the study provided valuable insights, it had limitations, including the heterogeneity of subjects and the reliance on a single inflammatory biomarker. The present study highlights the association of elevated hs-CRP with dyslipidemia and micronutrient deficiencies. These would extend our understanding of the mechanism of lesion progression in CAD and the association of micronutrients, particularly vitamins and minerals with anti-inflammatory properties. Future research should consider these limitations and explore the long-term inflammatory responses and mechanisms of lesion progression in cardiovascular disease.

## Conclusions

In conclusion, the present study examined the association between hs-CRP, lipid panel, and various vital micronutrients in subjects categorized based on CRP levels. The large sample size of the study strengthens its findings and allows for the detection of possible associations within these categories. The study revealed significant associations between hs-CRP and lipid parameters, such as cholesterol, LDL, and triglycerides, within the CRP categories. These associations indicate that elevated levels of cholesterol and triglycerides may be linked to inflammatory responses, as evidenced by higher hs-CRP levels in these groups. Conversely, a strong negative correlation was observed between hs-CRP and HDL, which aligns with previous studies reporting an inverse association between HDL and systemic inflammatory markers.

Additionally, the study demonstrated associations between hs-CRP and various micronutrients, including vitamin A, vitamin B9 (folate), vitamin D3, and vitamin E, within the categorized groups. These findings highlight the complex interplay between lipid levels, micronutrients, and inflammation. By categorizing subjects based on CRP levels, our analysis provides insights into the specific associations between hs-CRP and lipid and micronutrient profiles, contributing to a deeper understanding of inflammatory processes and their nutritional influences.
